# Identification of a Chromosome 1 Substitution Line B6-Chr1BLD as a Novel Hyperlipidemia Model *via* Phenotyping Screening

**DOI:** 10.3390/metabo12121276

**Published:** 2022-12-16

**Authors:** Xu Li, Minli Sun, Hao Qi, Cunxiang Ju, Zhong Chen, Xiang Gao, Zhaoyu Lin

**Affiliations:** 1 State Key Laboratory of Pharmaceutical Biotechnology, MOE Key Laboratory of Model Animals for Disease Study, Jiangsu Key Laboratory of Molecular Medicine, Model Animal Research Center, National Resource Center for Mutant Mice of China, Nanjing Drum Tower Hospital, School of Medicine, Nanjing University, Nanjing 210061, China; 2 GemPharmatech Inc., 12 Xuefu Road, Jiangbei New Area, Nanjing 210061, China

**Keywords:** hyperlipidemia model, chromosome 1 substitution lines, lipid metabolism

## Abstract

Hyperlipidemia is a chronic disease that seriously affects human health. Due to the fact that traditional animal models cannot fully mimic hyperlipidemia in humans, new animal models are urgently needed for basic drug research on hyperlipidemia. Previous studies have demonstrated that the genomic diversity of the wild mice chromosome 1 substitution lines was significantly different from that of laboratory mice, suggesting that it might be accompanied by phenotypic diversity. We first screened the blood lipid-related phenotype of chromosome 1 substitution lines. We found that the male HFD-fed B6-Chr1BLD mice showed more severe hyperlipidemia-related phenotypes in body weight, lipid metabolism and liver lesions. By RNA sequencing and whole-genome sequencing results of B6-Chr1BLD, we found that several differentially expressed single nucleotide polymorphism enriched genes were associated with lipid metabolism-related pathways. Lipid metabolism-related genes, mainly including *Aida*, *Soat1*, *Scly* and *Ildr2,* might play an initial and upstream role in the abnormal metabolic phenotype of male B6-Chr1BLD mice. Taken together, male B6-Chr1BLD mice could serve as a novel, polygenic interaction-based hyperlipidemia model. This study could provide a novel animal model for accurate clinical diagnosis and precise medicine of hyperlipidemia.

## 1. Introduction

Hyperlipidemia is a disease caused by elevated serum levels of various lipid metabolism-related factors, mainly including total cholesterol (CHO), triglycerides (TG), and low-density lipoprotein cholesterol (LDL-C) [1]. Many studies have shown that hyperlipidemia could induce multiple complications and cause serious damage to human health. Hyperlipidemia aggravates glomerulopathy and participates in the pathological process of human chronic kidney disease [2,3,4]. The accumulation of plasma LDL-C significantly increases the risk of atherosclerotic cardiovascular disease [5,6]. The TG/HDL ratio could be used as a risk indicator for judging the progression of atherosclerosis in prediabetes [7]. Blood lipid disorder also promotes the development of tumors and increases the mortality of cancer patients [8,9,10]. High-fat diet (HFD) exacerbates the disease phenotype of septic mice compared with chow diet (CD)-fed mice [11]. Taken together, hyperlipidemia is a complex disease co-related with metabolism and immunity.

Current hyperlipidemia animal models mainly include the HFD induction model and the classical genetic mutation induction model. HFD-induced hyperlipidemia models have been used to study the pathological process of many complicated diseases, such as diabetes, colorectal cancer, non-alcoholic fatty liver disease and atherosclerosis [12,13,14,15]. Lipoprotein lipase gene-deficient mice cause severe hypertriglyceridemia [16,17]. Apolipoprotein E-deficient mice are also typical models of hyperlipidemia and have been used in the research of multiple diseases [18,19,20]. Human hyperlipidemia is a complex pathological process that is caused by multiple genetic and environmental factors. Based on laboratory mice, the HFD induction model and the single genetic alteration model could not fully mimic human hyperlipidemia. Therefore, new hyperlipidemia animal models are urgently needed for the study of human hyperlipidemia.

The chromosome of the substitution lines is replaced by the corresponding single complete chromosome of the donor mouse, which helps to identify the function of specific chromosomes and obtain diverse phenotypes [21,22,23]. Compared to the laboratory lines, the wild mice have more abundant genetic polymorphisms and could serve as better donor mice [24,25]. Chromosome 1 (Chr 1) contains multiple gene loci related to blood lipid metabolism [26,27,28,29,30]. Professor Xiao’s group constructed new lines named Chr 1 substitution lines with wild mice from different areas of China [31,32,33,34,35]. Hybrid embryos were implanted into specific pathogen-free foster mothers, and the F1 hybrids were backcrossed to the recipient mice to obtain sufficient Chr 1 substitution mice for future study [31]. Whole genome sequencing (WGS) results revealed that Chr 1 substitution lines had abundant SNP sites. The levels of lipid metabolism-related factors in the blood also showed significant differences between Chr 1 substitution lines and control lines [32]. Chr 1 substitution lines showed differences in genes related to behavior, weight, and immunity [33]. Chr 1 substitution line B6-Chr1^KM^ contained unique genetic variants, and several genes were associated with different human diseases [34]. The liver expression profile results revealed significant changes in lipid metabolism-related pathways in the Chr 1 substitution lines [35]. Taken together, Chr 1 substitution lines might have the potential as the hyperlipidemia model to study human dyslipidemia-related diseases.

In this study, we investigated then checked the major hyperlipidemia-related phenotypes of four Chr 1 substitution lines, including B6/JGpt-Chr1SJ(jhxiao)/Gpt (B6-Chr1SJ), B6/JGpt-Chr1LY(jhxiao)/Gpt (B6-Chr1LY), B6/JGpt-Chr1ZZ2(jhxiao)/Gpt (B6-Chr1ZZ2) and B6/JGpt-Chr1BLD(jhxiao)/Gpt (B6-Chr1BLD). The Chr 1 substitution line with the most severe hyperlipidemia phenotype was chosen to further genomic analysis via RNA sequencing and WGS. This study will identify suitable hyperlipidemia mouse models through phenotypic analysis and uncover hyperlipidemia-related candidate genes via multi-omics analysis.

## 2. Materials and Methods

### 2.1. Animal Studies

C57BL/6J mice, B6-Chr1SJ mice, B6-Chr1LY mice, B6-Chr1ZZ2 mice and B6-Chr1BLD mice were provided by GemPharmatech (Nanjing, China). All mice were housed in a specific pathogen-free facility accredited by the Association for Assessment and Accreditation of Laboratory Animal Care International. All animal welfare and experimental procedures were approved by the Animal Care and Use Committee of the Model Animal Research Center, Nanjing University (Nanjing, China). The mice were maintained on a 12/12 h light/dark cycle at 21–25 °C and 40–70% humidity with access to sterile pellet food and water ad libitum. Animals were labeled by ear numbers throughout the experimental period, and each animal had a unique animal number. The mice were fed with a high-fat diet (HFD) (D12451, 45% kcal% fat; Research diet, New Brunswick, NJ, USA) for indicated weeks. Body weights were measured at the indicated ages during the entire experimental period.

### 2.2. Experimental Design

8-week-old B6 and B6-Chr1BLD mice were fed with a high-fat diet (HFD) (D12451, 45% kcal% fat; Research diet, New Brunswick, NJ, USA) for 25 weeks. Mice were sacrificed, and the tissue samples of the liver and brain were collected for transcriptomics analysis and SNP analysis.

### 2.3. Serum Analysis

Mice were first put into an induction chamber to anesthetize the mouse through inhalation anesthesia with isoflurane (R510-22-10, RWD). And then, the blood of mice was collected from orbit into 1.5-mL EDTA-coated Eppendorf tubes (Eppendorf, 0030125150) containing 8 uL of 0.5 M EDTA. The serum was separated from the whole blood of mice by centrifugation at 6000 rpm for 10 min. The serum levels of TG, CHO, HDL-C and LDL-C were measured by AUTOMATIC ANALYZER (HITACHI 7020, Japan Care Co., Ltd. Osaka, Japan) and according to the manufacturer’s instructions. Mice were euthanized by CO_2_ (flow rate as 3 L/min), and CO_2_ was continued until 1 min after breathing stopped. Euthanasia was confirmed after stopping CO_2_.

### 2.4. H&E Staining

The liver tissues were harvested from mice and then fixed in 4% paraformaldehyde (Solarbio, P1110). The tissues were dehydrated by an Automatic dehydrator (Leica ASP 200S) and were embedded into paraffin wax blocks (Merck, 1.07150) by a paraffin embedding machine (Leica EG1150). The liver tissue samples were cut into 5 μm sections by a paraffin slicing machine (Leica RM2235). The liver sections were added into Xylene (Sinopharm Chemical Reagent, 10023418) for dewaxing and went through rehydration with alcohol (Merck, 1012772) before stain nuclei with hematoxylin (BASO, C201002). The liver sections were dehydrated with alcohol before being stained with eosin (BASO, C201201) for cytoplasm. Finally, dehydration with alcohol and xylene, followed by sealing with neutral resin (Solarbio, G8590). 

### 2.5. Oil Red O Staining

The liver tissues were harvested from mice and then fixed in 4% paraformaldehyde (Solarbio, P1110). OTC (Solarbio, 4583) embedding was performed after dehydration with 40% sucrose (Sigma, V900116). The frozen liver tissue samples were cut into 10 μm sections (Leica, German/HistoCore AUTOCUT+ HistoCore) and air dry at RT. Rinse briefly with ddH_2_O to remove OTC and air dry at RT. A few dips in 60% isopropyl alcohol (Merck, W292907) for 2–5 min and oil-red O (Merck, O1391) working solution for 15 min. Rinse with four changes of ddH_2_O for 5 × 4 min. Then counter with hematoxylin (BASO, C201002) for 2 min. Finally, rinse with four changes of distilled water and mount with glycerol jelly 50% (Phygene, PH1425).

### 2.6. Transcriptomics Analysis

RNA from the liver tissues was isolated according to the instructions of the kits. RNAiso Plus (#9109, TAKARA) was used for this process. RNA quality was detected after extraction. Illumina NovaSeq 6000 (Illumina, San Diego, CA, USA) and PE 150 were used for sequencing. The raw reads were filtered by removing reads containing adapter, ploy-N, and low-quality reads for subsequent analysis. Then paired-end clean reads were aligned to the reference genome using Hisat2 (v2.0.5) [36,37]. The mapped reads of each sample were assembled using Stringtie (v1.3.3) [36,38] with a reference-based approach. Cuffdiff (v1.3.0) [39] was used to calculate FPKMs (fragments per kilobase of exon per million fragments mapped) for coding genes in each sample. The FPKMs were computed by summing the FPKMs of the transcripts in each gene group. Cuffdiff provides statistical routines for determining differential expression in digital transcript or gene expression datasets using a model based on a negative binomial distribution. Genes with corrected *p*-value less than 0.05 and the absolute value of log2 (fold change) greater than or equal to 1 were assigned as significantly differentially expressed. Metascape [40] was used to further analyze significantly expressed genes by performing enrichment analysis.

### 2.7. SNP Analysis

DNA from the brain tissues was isolated according to the proteinase K DNA extraction method. DNA quality was detected after extraction. Illumina NovaSeq 6000 and PE 150 were used for sequencing. Clean reads were obtained by removing reads with adapters, reads in which unknown bases were more than 10% and low-quality reads. BWA (Burrows-Wheeler Aligner, v0.7.13-r1126) [41] was used to align the clean reads of each sample against the reference genome with default parameters. Alignment files were converted to BAM files using SAMtools software (v 1.3, settings: view -Sb -T) [42]. Variant calling was performed for all samples by using the HaplotypeCaller in GATK software [43]. ANNOVAR [44], an efficient software tool, was used to annotate SNP based on the gene model annotation files for the reference genome. Genes with more than 3 exonic non-synonymous or stopgain SNPs enriched were assigned as significantly mutant. Metascape was used to further analyze significant mutant genes by performing enrichment analysis.

### 2.8. Statistics

GraphPad Prism 8.0.1 (GraphPad Software, San Diego, CA, USA) was used to quantize body weight, serum analysis, volcano plot and functional annotation SNPs data. All data were analyzed statistically by student *t*-test. * *p* < 0.05, ** *p* < 0.01, *** *p* < 0.001, **** *p* < 0.0001, ns = not significant.

## 3. Results

### 3.1. HFD-fed B6-Chr1SJ Line Showed Significant Abnormalities in Body Weight, Lipid Metabolism and Liver Lesions

**Figure 1:**
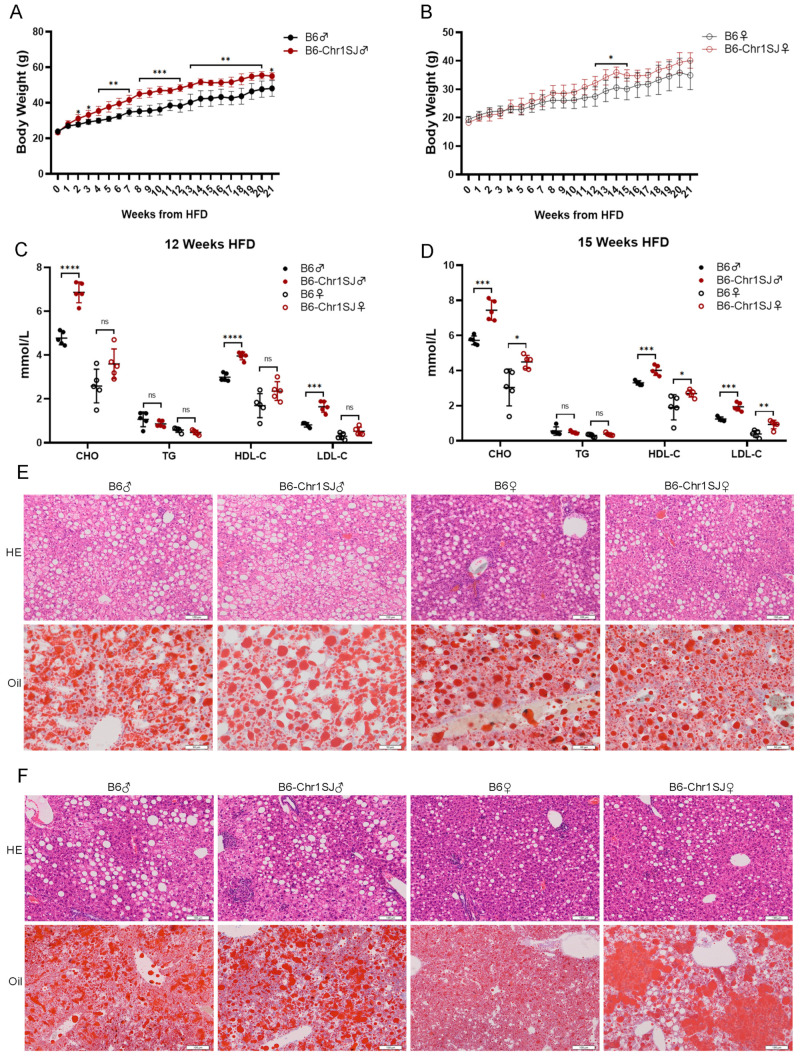
HFD-fed B6-Chr1SJ line showed significant abnormalities in body weight, lipid metabolism and liver lesions. (**A**) Average body weight trajectory in HFD-fed male mice (*n* = 5 per group). (**B**) Average body weight trajectory in HFD-fed female mice (*n* = 4–5 per group). (**C**) The serum lipid levels (CHO, TG, HDL-C, and LDL-C) of 20-week-old C57BL/6J and B6-Chr1SJ mice (HFD fed for 12 weeks) (*n* = 5 per group). (**D**) The serum lipid levels (CHO, TG, HDL-C, and LDL-C) of 23-week-old C57BL/6J and B6-Chr1SJ mice (HFD fed for 15 weeks) (*n* = 5 per group). (**E**) The representative H&E and Oil Red O staining in liver sections of 36-week-old C57BL/6J and B6-Chr1SJ mice (HFD fed for 28 weeks). Scale bars (H&E staining) = 100 μm. Scale bars (Oil Red O staining) = 50 μm. (**F**) The representative H&E and Oil Red O staining in liver sections of 43-week-old C57BL/6J and B6-Chr1SJ mice (HFD fed for 35 weeks). Scale bars (H&E staining) = 100 μm. Scale bars (Oil Red O staining) = 100 μm. (**A**–**D**) Data are presented as the mean ± SD. *T*-test: * *p* < 0.05, ** *p* < 0.01, *** *p* < 0.001, **** *p* < 0.0001. ns = not significant. HE: hematoxylin-eosin staining. Oil: Oil Red O staining. CHO: total cholesterol. TG: triglycerides. HDL-C: high-density lipoprotein cholesterol. LDL-C: low-density lipoprotein cholesterol. ♀: female. ♂: male.

To screen out the suitable hyperlipidemia mouse model, we explored the hyperlipidemia-related phenotypes in four Chr 1 substitution lines under HFD feeding. The body weight of the male HFD-fed B6-Chr1SJ mice was significantly higher than the control group after 2 weeks of HFD, and the body weight of female HFD-fed B6-Chr1SJ mice was significantly upregulated only between 12 to 15 weeks’ HFD (Figure 1A,B). The serum levels of lipid metabolism-related factors, including CHO, TG, high-density lipoprotein cholesterol (HDL-C) and LDL-C, were further detected. Elevated serum levels of CHO, TG and LDL-C and reduced HDL-C could worsen hyperlipidemia [1,45]. Consistently, the serum levels of CHO, HDL-C and LDL-C in male B6-Chr1SJ mice were significantly higher than control male mice after 12-week HFD, but there was no significant difference between the female mice, suggesting that male B6-Chr1SJ mice had a more severe hyperlipidemia phenotype (Figure 1C). The serum levels of CHO, HDL-C and LDL-C in both male and female B6-Chr1SJ mice were significantly higher than control mice at 15-week HFD (Figure 1D). Additionally, we analyzed the hepatic pathological changes by H&E and Oil Red O staining [46,47,48]. H&E staining showed that the levels of inflammatory cell infiltration were not significantly different between the B6-Chr1SJ lines and control mice at 28-week HFD. Oil Red O staining confirmed that the levels of lipid accumulation were similar in these two groups at 28-week HFD (Figure 1E). However, the results of H&E staining and Oil Red O staining showed that only the livers of female B6-Chr1SJ mice developed more severe lesions than control mice at 35-week HFD (Figure 1F). In summary, the male HFD-fed B6-Chr1SJ mice showed significant abnormalities in body weight and lipid metabolism, and the female HFD-fed B6-Chr1SJ mice showed significant abnormalities in body weight, lipid metabolism and liver lesions. The female HFD-fed B6-Chr1SJ mice might serve as a hyperlipidemia mouse model.

### 3.2. HFD-fed B6-Chr1LY Line Showed Significant Abnormalities in Body Weight, Lipid Metabolism and Liver Lesions

**Figure 2:**
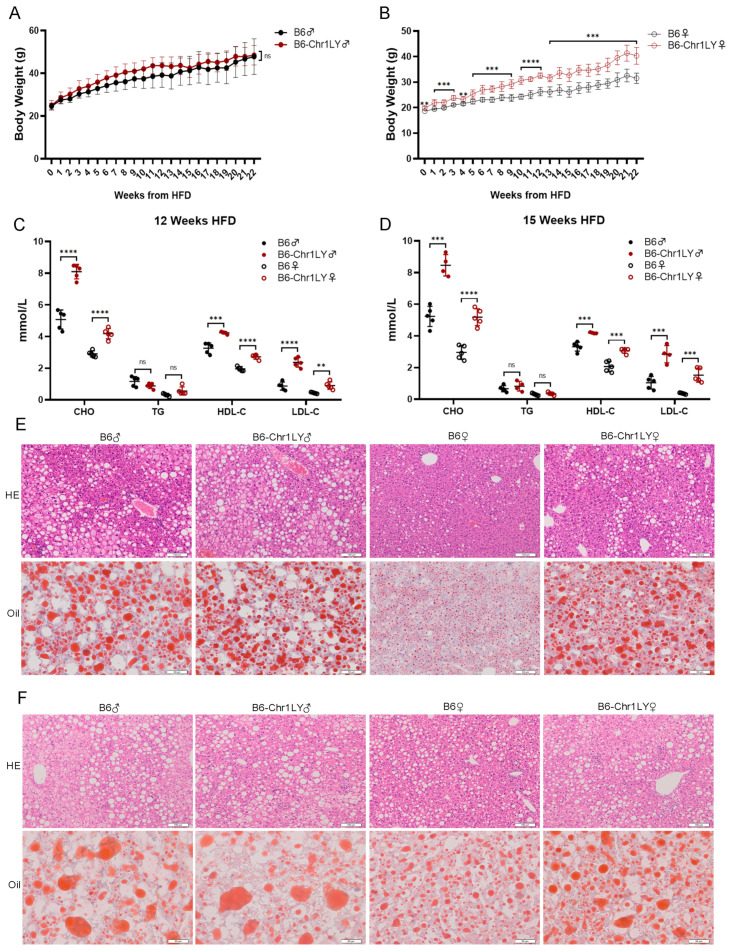
HFD-fed B6-Chr1LY line showed significant abnormalities in body weight, lipid metabolism and liver lesions. (**A**) Average body weight trajectory in HFD-fed male mice (*n* = 4–5 per group). (**B**) Average body weight trajectory in HFD-fed female mice (*n* = 5 per group). (**C**) The serum lipid levels (CHO, TG, HDL-C, and LDL-C) of 20-week-old C57BL/6J and B6-Chr1LY mice (HFD fed for 12 weeks) (*n* = 5 per group). (**D**) The serum lipid levels (CHO, TG, HDL-C, and LDL-C) of 23-week-old C57BL/6J and B6-Chr1LY mice (HFD fed for 15 weeks) (*n* = 4–5 per group). (**E**) The representative H&E and Oil Red O staining in liver sections of 30-week-old C57BL/6J and B6-Chr1LY mice (HFD fed for 22 weeks). Scale bars (H&E staining) = 100 μm. Scale bars (Oil Red O staining) = 50 μm. (**F**) The representative H&E and Oil Red O staining in liver sections of 50-week-old C57BL/6J and B6-Chr1LY mice (HFD fed for 42 weeks). Scale bars (H&E staining) = 100 μm. Scale bars (Oil Red O staining) = 50 μm. (**A**–**D**) Data are presented as the mean ± SD. *T*-test: ** *p* < 0.01, *** *p* < 0.001, **** *p* < 0.0001. ns = not significant. HE: hematoxylin-eosin staining. Oil: Oil Red O staining. CHO: total cholesterol. TG: triglycerides. HDL-C: high-density lipoprotein cholesterol. LDL-C: low-density lipoprotein cholesterol. ♀: female. ♂: male.

The body weight of the female HFD-fed B6-Chr1LY mice was significantly higher than the control group after one week of HFD, but there was no significant difference between the male mice (Figure 2A,B). The serum levels of CHO, HDL-C and LDL-C in both male and female B6-Chr1LY mice were significantly higher than in control mice at 12-week HFD and 15-week HFD (Figure 2C,D). The results of H&E staining and Oil Red O staining indicated that the liver sections of female B6-Chr1LY mice had more severe lesions at 22-week HFD and 42-week HFD compared with control female mice, but there was no more severe liver inflammation and lipid accumulation in male B6-Chr1LY mice (Figure 2E,F). All these results suggest that the male HFD-fed B6-Chr1LY mice showed significant abnormalities only in lipid metabolism, and the female HFD-fed B6-Chr1LY mice showed significant abnormalities in body weight, lipid metabolism and liver lesions. In summary, the female HFD-fed B6-Chr1LY mice might serve as a hyperlipidemia mouse model. 

### 3.3. HFD-fed B6-Chr1ZZ2 Line Showed Significant Abnormalities in Body Weight, Lipid Metabolism and Liver Lesions

**Figure 3:**
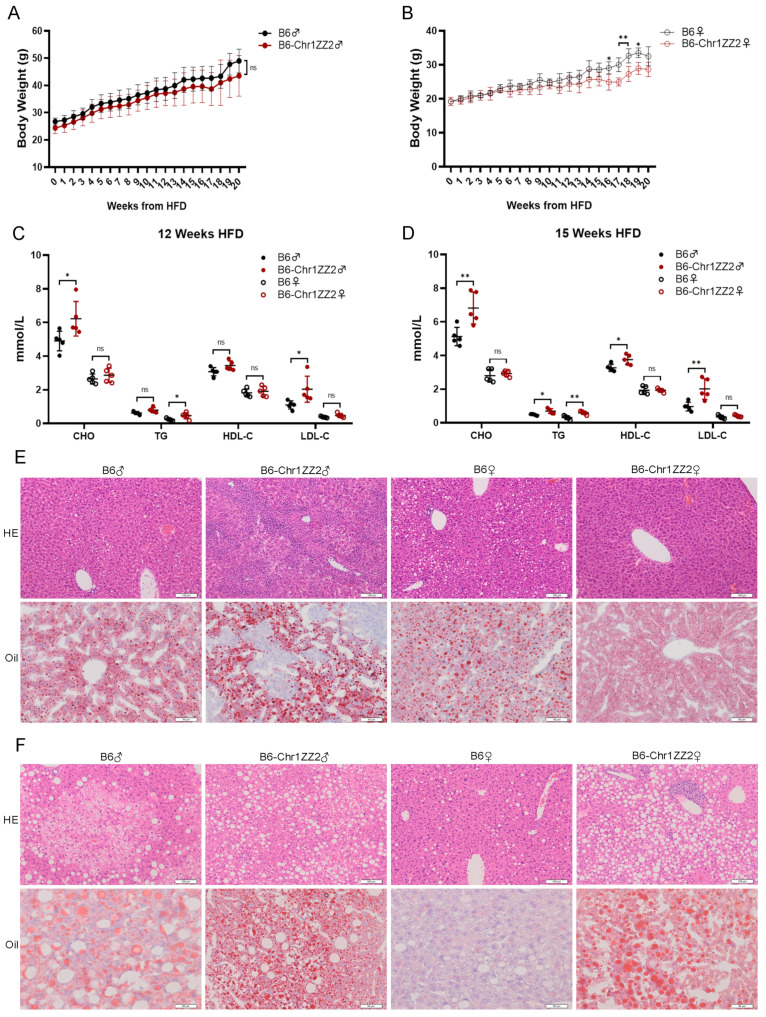
HFD-fed B6-Chr1ZZ2 line showed significant abnormalities in body weight, lipid metabolism and liver lesions. (**A**) Average body weight trajectory in HFD-fed male mice (*n* = 3–5 per group). (**B**) Average body weight trajectory in HFD-fed female mice (*n* = 3–5 per group). (**C**) The serum lipid levels (CHO, TG, HDL-C, and LDL-C) of 20-week-old C57BL/6J and B6-Chr1ZZ2 mice (HFD fed for 12 weeks) (*n* = 5 per group). (**D**) The serum lipid levels (CHO, TG, HDL-C, and LDL-C) of 23-week-old C57BL/6J and B6-Chr1ZZ2 mice (HFD fed for 15 weeks) (*n* = 5 per group). (**E**) The representative H&E and Oil Red O staining in liver sections of 27-week-old C57BL/6J and B6-Chr1ZZ2 mice (HFD fed for 19 weeks). Scale bars (H&E staining) = 100 μm. Scale bars (Oil Red O staining) = 50 μm. (**F**) The representative H&E and Oil Red O staining in liver sections of 40-week-old C57BL/6J and B6-Chr1ZZ2 mice (HFD fed for 32 weeks). Scale bars (H&E staining) = 100 μm. Scale bars (Oil Red O staining) = 50 μm. (**A**–**D**) Data are presented as the mean ± SD. *T*-test: * *p* < 0.05, ** *p* < 0.01. ns = not significant. HE: hematoxylin-eosin staining. Oil: Oil Red O staining. CHO: total cholesterol. TG: triglycerides. HDL-C: high-density lipoprotein cholesterol. LDL-C: low-density lipoprotein cholesterol. ♀: female. ♂: male.

The body weight of the female HFD-fed B6-Chr1ZZ2 mice was significantly higher than the control group, only between 16 to 19 weeks’ HFD, but there was no significant difference between the male mice (Figure 3A,B). The serum levels of CHO and LDL-C in male B6-Chr1ZZ2 mice were significantly higher than in control mice at 12 weeks HFD (Figure 3C). The serum levels of TG and HDL-C in male B6-Chr1ZZ2 mice were also significantly upregulated at 15-week HFD (Figure 3D). As for female B6-Chr1ZZ2 mice, only the serum levels of TG were significantly higher than female control mice at both 12-week HFD and 15-week HFD (Figure 3C,D). The results of H&E staining and Oil Red O staining indicated that the liver sections of male and female B6-Chr1ZZ2 mice had more severe lesions only at 32-week HFD compared with control mice (Figure 3E,F). Taken together, the male HFD-fed B6-Chr1ZZ2 mice showed significant abnormalities only in lipid metabolism and liver lesions. The female HFD-fed B6-Chr1ZZ2 mice showed significant abnormalities in body weight, lipid metabolism and liver lesions and might serve as a hyperlipidemia mouse model.

### 3.4. HFD-fed B6-Chr1BLD Line Showed Significant Abnormalities in Body Weight, Lipid Metabolism and Liver Lesions

**Figure 4:**
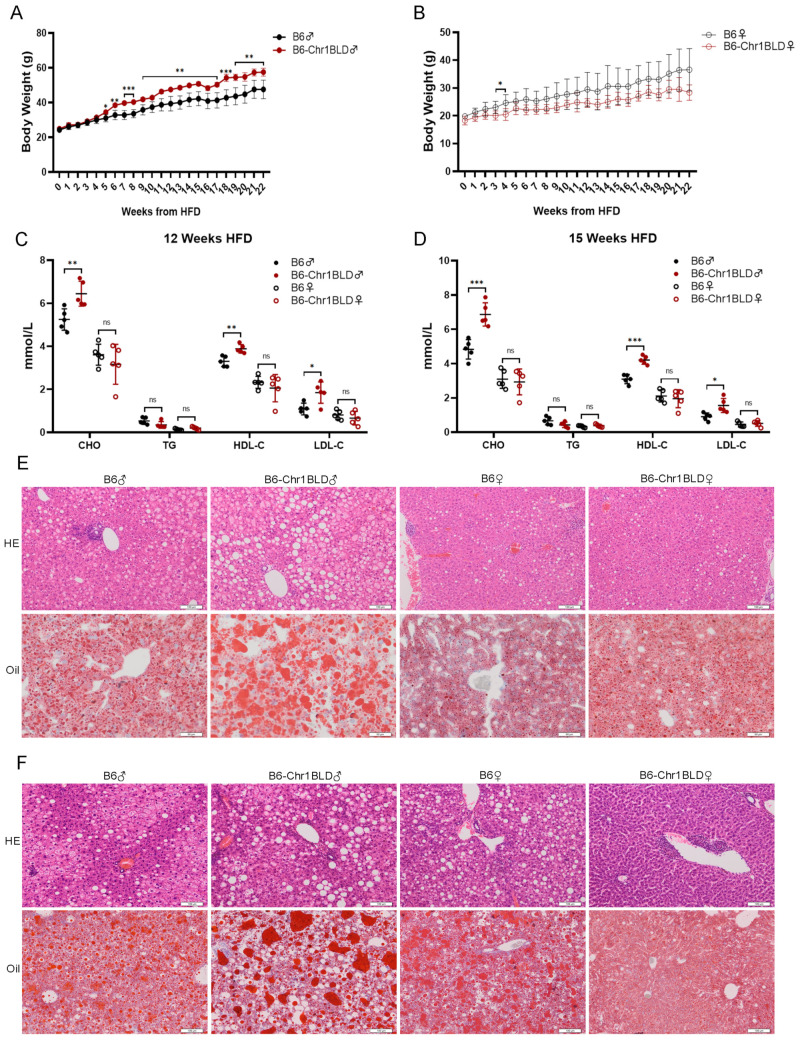
HFD-fed B6-Chr1BLD line showed significant abnormalities in body weight, lipid metabolism and liver lesions. (**A**) Average body weight trajectory in HFD-fed male mice (*n* = 5 per group). (**B**) Average body weight trajectory in HFD-fed female mice (*n* = 5 per group). (**C**) The serum lipid levels (CHO, TG, HDL-C, and LDL-C) of 20-week-old C57BL/6J and B6-Chr1BLD mice (HFD fed for 12 weeks) (*n* = 5 per group). (**D**) The serum lipid levels (CHO, TG, HDL-C, and LDL-C) of 23-week-old C57BL/6J and B6-Chr1BLD mice (HFD fed for 15 weeks) (*n* = 5 per group). (**E**) The representative H&E and Oil Red O staining in liver sections of 34-week-old C57BL/6J and B6-Chr1BLD mice (HFD fed for 26 weeks). Scale bars (H&E staining) = 100 μm. Scale bars (Oil Red O staining) = 50 μm. (**F**) The representative H&E and Oil Red O staining in liver sections of 45-week-old C57BL/6J and B6-Chr1BLD mice (HFD fed for 37 weeks). Scale bars (H&E staining) = 100 μm. Scale bars (Oil Red O staining) = 100 μm. (**A**–**D**) Data are presented as the mean ± SD. *T*-test: * *p* < 0.05, ** *p* < 0.01, *** *p* < 0.001. ns = not significant. HE: hematoxylin-eosin staining. Oil: Oil Red O staining. CHO: total cholesterol. TG: triglycerides. HDL-C: high-density lipoprotein cholesterol. LDL-C: low-density lipoprotein cholesterol. ♀: female. ♂: male.

The body weight of the male HFD-fed B6-Chr1BLD mice was significantly higher than the control group after 5 weeks of HFD, but the body weight of female B6-Chr1BLD mice was significantly upregulated only between 3 to 4 weeks of HFD (Figure 4A,B). The serum levels of CHO, HDL-C and LDL-C in male B6-Chr1BLD mice were significantly higher than in control mice at both 12-week HFD and 15-week HFD, but there was no significant difference between the female mice (Figure 4C,D). The results of H&E staining and Oil Red O staining showed that the liver sections of male B6-Chr1BLD mice had more severe lesions at both 26-week HFD and 37-week HFD compared with control mice, but there was no more severe liver inflammation and lipid accumulation in female B6-Chr1BLD mice (Figure 4E,F). In summary, the female HFD-fed B6-Chr1BLD mice showed severe phenotype only in body weight. The male HFD-fed B6-Chr1BLD mice showed more severe hyperlipidemia-related phenotypes in body weight, lipid metabolism and liver lesions and might serve as a hyperlipidemia mouse model.

### 3.5. Single Nucleotide Polymorphism (SNP) Analysis and Transcriptomics Analysis of B6-Chr1BLD Mice

Based on the above results, the female B6-Chr1SJ mice, the female B6-Chr1LY mice, the female B6-Chr1ZZ2 mice and the male B6-Chr1BLD mice showed more severe hyperlipidemia-related phenotypes in body weight, the serum levels of lipid metabolism-related factors and hepatic pathological phenotypes, and they might serve as hyperlipidemia mouse models (Figure 1, Figure 2, Figure 3 and Figure 4). In the elderly population, the proportion of hyperlipidemia is higher in women than that of men, while in the young and middle-aged population, the incidence, and the harm of hyperlipidemia in men are generally higher than that of women [49,50]. Our data only revealed hyperlipidemia-related phenotypes in young and middle-aged mice (Figure 1, Figure 2, Figure 3 and Figure 4). We need to further identify the relevant metabolism phenotypes in older mice to verify whether female B6-Chr1SJ mice, B6-Chr1LY mice and B6-Chr1ZZ2 mice could be used as models for female patients. 

**Figure 5:**
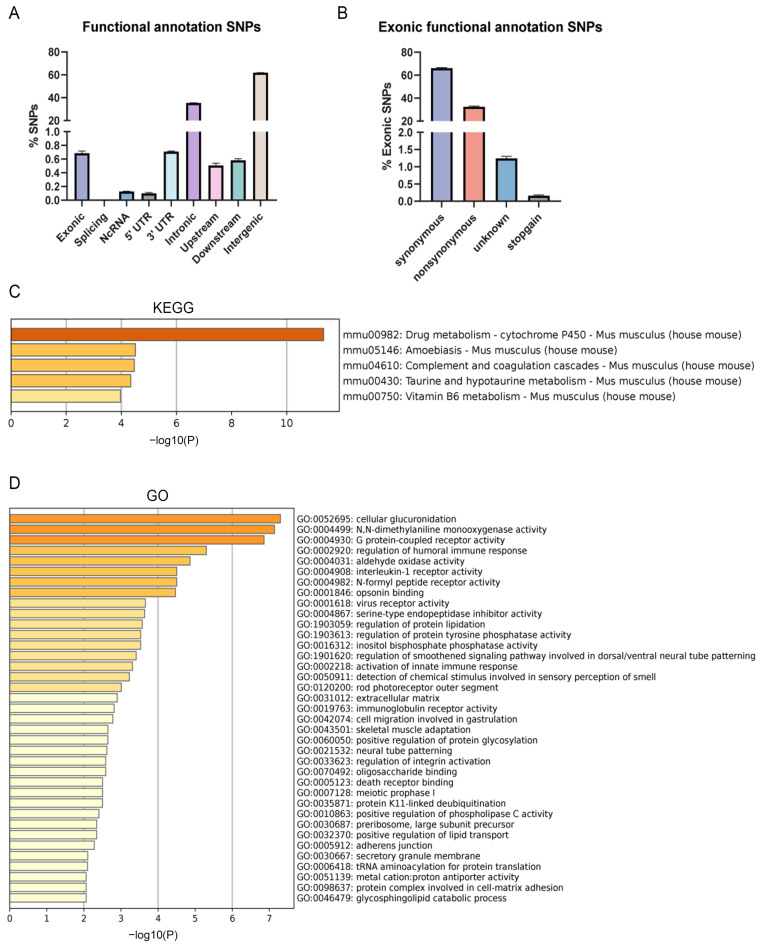
SNP analysis of B6-Chr1BLD mice. (**A**) Distribution of functional consequences of SNPs. (**B**) Distribution of types of SNPs (synonymous and non-synonymous) in the exonic region. Non-synonymous SNPs and stopgain SNPs were used for further SNP enrichment analysis. (**C**) Enriched KEGG terms. (**D**) Enriched GO terms.

Therefore, we next choose the B6-Chr1BLD line for further research. The brain was used for WGS to identify SNPs-enriched genes and their function. SNP analysis showed the distribution patterns of SNPs in various genomic regions. A large number of SNPs were identified within the intergenic region (~61.8%) and intronic region (~35.4%), whereas there were only about 0.69% SNPs in the exonic region (Figure 5A). A low percentage of SNPs were identified in the splicing region (~0.001%), non-coding RNA (ncRNA) region (~0.13%), 5′ UTR region (~0.1%), 3′ UTR region (~0.71%), upstream region (~0.51%) and downstream region (~0.58%). SNPs in the exonic region were further classified into synonymous SNPs (~66.1%), non-synonymous SNPs (32.5%), unknown SNPs (1.2%) and stopgain SNPs (~0.2%) (Figure 5B). Genes containing non-synonymous SNPs and stopgain SNPs were further analyzed. KEGG analysis showed that a total of 5 pathways were significantly enriched (Figure 5C). Pathways associated with metabolism (drug metabolism-cytochrome P450, Taurine and hypotaurine metabolism and Vitamin B6 metabolism) were enriched. Gene ontology (GO) analysis showed that a total of 37 GO terms were significantly enriched, including regulation of the lipoprotein metabolic process and lipid transport (Figure 5D). 

**Figure 6:**
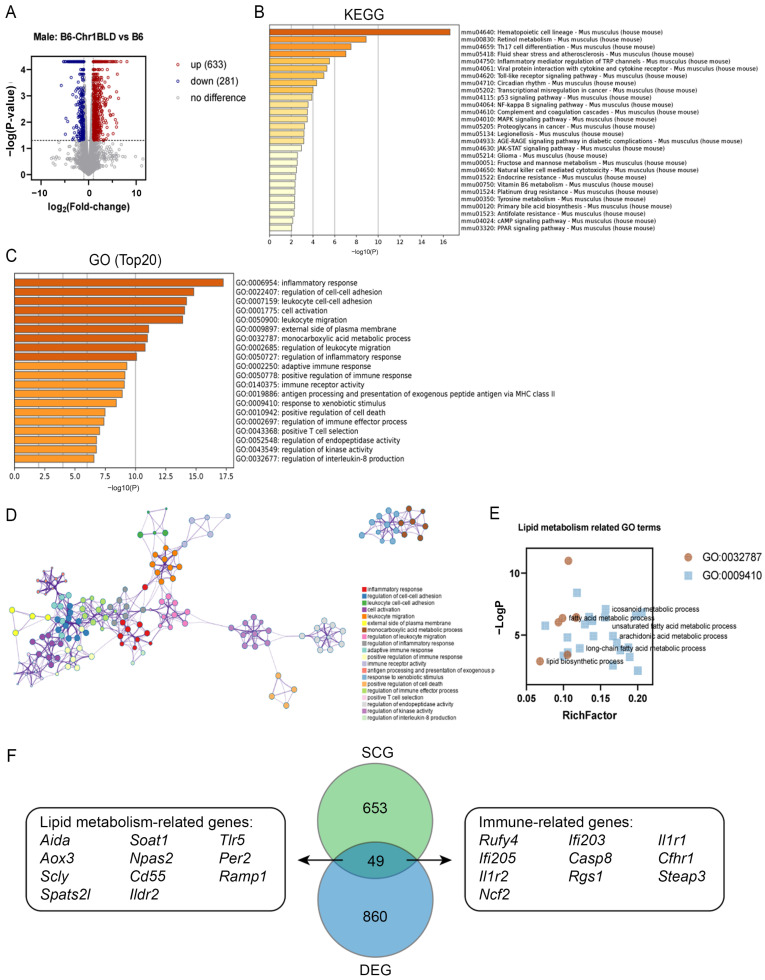
Transcriptome analysis of livers from male B6-Chr1BLD mice. (**A**) Volcano plot of DEGs from male B6-Chr1BLD mice and male B6 mice. (**B**) Enriched KEGG terms. (**C**) Top 20 enriched GO terms. (**D**) Enriched ontology clusters colored by cluster ID. (**E**) Lipid metabolism-related GO terms. (**F**) A Venn diagram showing the overlap between SCGs and DEGs. Lipid metabolism and immune-related genes are shown.

Furthermore, liver tissue samples from B6-Chr1BLD mice and control mice after 25-week HFD were used for expression profile analysis. The volcano plot showed that a total of 914 differentially expressed genes (DEGs) were identified, including 633 upregulated genes and 281 down-regulated genes (Figure 6A). KEGG analysis showed that a total of 28 pathways were significantly enriched by DEGs (Figure 6B). Pathways associated with lipid metabolism (such as primary bile acid biosynthesis and PPAR signaling pathway), cardiovascular disease (fluid shear stress and atherosclerosis), and endocrine and metabolic disease (AGE-RAGE signaling pathway in diabetic complications) were enriched. Furthermore, more severe levels of obesity might cause increased inflammation, which leads to many immune-related pathways being enriched. GO analysis showed that DEGs were enriched in lipid metabolism-related terms such as fatty acid metabolic process, lipid biosynthetic process, icosanoid metabolic process, unsaturated fatty acid metabolic process, arachidonic acid metabolic process and long-chain fatty acid metabolic process in addition to GO terms related to immune response (Figure 6C–E). To explore the correlation between SNPs-containing genes (SCGs) and DEGs, we used a Venn diagram to show the overlap between them (Figure 6F). The 49 shared genes on Chr1 contained 11 genes that had been reported to be associated with lipid metabolism, including *Aida*, *Soat1*, *Tlr5*, *Aox3*, *Npas2*, *Per2*, *Scly*, *Cd55*, *Ramp1*, *Spats2l* and *Ildr2* [51,52,53,54,55,56,57,58,59,60,61,62]. The previous study showed that *Aida*^−/−^ mice grew under thermal-neutral conditions or HFD feeding displayed increased intestinal fatty acid re-esterification, circulating and tissue triacylglycerol, accompanied by a severe increase in adiposity without enhanced adipogenesis [60]. The deficiency of SOAT1 alleviated HFD-induced and spontaneous atherosclerotic lesions in ApoE−/− mice, accompanied by decreased TG, total cholesterol (TC) and LDL-C, and enhanced HDL-C in serum of ApoE^−/−^ mice [61,62]. *Scly*^−/−^ mice showed increased susceptibility to metabolic syndrome and diet-induced obesity [55]. *Ildr2* knockdown mice showed hepatic steatosis, with increased hepatic and circulating TG and CHO in the liver [59]. All of these lipid metabolism-related genes might play an initial and upstream role in the abnormal metabolic phenotype of male B6-Chr1BLD mice. Furthermore, many previous studies demonstrated that hyperlipidemia-related diseases had intimately linked to immunity [63,64,65]. We found that there were 10 immune-related genes among the shared genes, including *Rufy4*, *Ifi203*, *Il1r1*, *Ifi205*, *Casp8*, *Cfhr1*, *Il1r2*, *Rgs1*, *Steap3* and *Ncf2* [66,67,68,69,70,71,72,73,74]. These genes may be involved in the regulatory process of hyperlipidemia in male B6-Chr1BLD mice.

Taken together, the male B6-Chr1BLD mice showed significant differences compared with control mice in lipid metabolism-related pathways and immune-related pathways in the analysis of both transcriptomics and genomics, which suggested that this substitution line had vast potential in the investigation of hyperlipidemia as a novel model.

## 4. Discussion

Hyperlipidemia is clinically divided into three main types, including the elevated TG type, the elevated CHO type, and the co-elevated TG and CHO type [75]. We identified four Chr 1 substitution lines based on the clinical classification. HFD-fed male and female B6-Chr1SJ line, male and female B6-Chr1LY line and male B6-Chr1BLD line belong to the elevated CHO type, female B6-Chr1ZZ2 line belongs to the elevated TG type, and male B6-Chr1ZZ2 line belongs to the co-elevated TG and CHO type (Figure 1, Figure 2, Figure 3 and Figure 4). Our Chr 1 substitution lines had diversity in hyperlipidemia types, which suggested they might have an advantage in the construction of the hyperlipidemia model and further precise drug screening.

Typical drugs for treating hyperlipidemia mainly target CHO (CHO-type) or target TG (TG-type) [76]. Based on the different mechanisms of drugs, the CHO-type drugs can be further classified into inhibiting HMG-CoA reductase (e.g., Statins), interrupting bile acid reabsorption (e.g., Bile acid sequestrants), and inhibiting intestinal absorption of cholesterol (e.g., Ezetimibe). The TG-type drugs mainly increase the activity of lipoprotein lipase (e.g., Fibrates). We would use these different typical drugs to verify whether our Chr 1 substitution lines will be sensitive to specific types of drugs and effectively alleviate the hyperlipidemic phenotype. Furthermore, these drugs have been reported to have many side effects, including damaging liver function and kidney function, causing skin rash, nausea, myopathy, polyneuropathy, and so on [76,77,78]. In the future, we would further develop new drugs for hyperlipidemia with fewer side effects based on the Chr 1 substitution lines. 

Hyperlipidemia can induce many complications, including non-alcoholic fatty liver disease, atherosclerosis, and diabetes mellitus [79,80,81,82]. The results of the liver pathology sections in the female B6-Chr1SJ line, female B6-Chr1LY line, male and female B6-Chr1ZZ2 line and male HFD-fed B6-Chr1BLD line showed that the levels of inflammatory infiltration and lipid accumulation of liver in these lines were significantly higher than those of the control mice (Figure 1F, Figure 2E,F, Figure 3F and Figure 4E,F). These results suggest that these lines might be used as the mouse model of hyperlipidemia-induced non-alcoholic fatty liver disease to study relevant pathological mechanisms and drug development. 

Additionally, we found that male B6-Chr1BLD mice may have potential research value in other diseases. The results of RNA sequencing and whole-genome sequencing in B6-Chr1BLD mice demonstrated that atherosclerosis, inflammatory responses, cancer, and glioma-related signaling pathways were enriched (Figure 5 and Figure 6). We found multiple immune-related genes among the shared genes between SCGs and DEGs (Figure 6F), which suggested that male B6-Chr1BLD mice might serve as a model for immunological diseases. We noticed that there were some important genes among the shared genes which might play key roles. Caspase-8 had been reported to be a protease with both pro-death and pro-survival functions, which mediated apoptosis induced by death receptors such as TNFR1, and inhibited necroptosis mediated by the kinase RIPK3 and the pseudokinase MLKL [55]. The ubiquitously expressed interleukin-1 receptor type 1 (IL-1R1) played vital roles in the innate immune system through interleukin-1-type cytokines including IL-1α, IL-1β and interleukin-1 receptor antagonist (IL-1Ra) [54]. RUFY4 was identified as a novel positive regulator that enhanced autophagic flux and induced drastic membrane redistribution and strongly tethered lysosomes when overexpressed [66].

Collectively, the male B6-Chr1BLD line might play an important role in cardiovascular and cerebrovascular disease models, as well as in models of cancer immune-related diseases. However, we only examined the basic blood lipid-related phenotypes, and we need to examine more molecular mechanisms to identify the potential of this Chr 1 substitution line as the related complication mouse model in the future.

**Figure 7:**
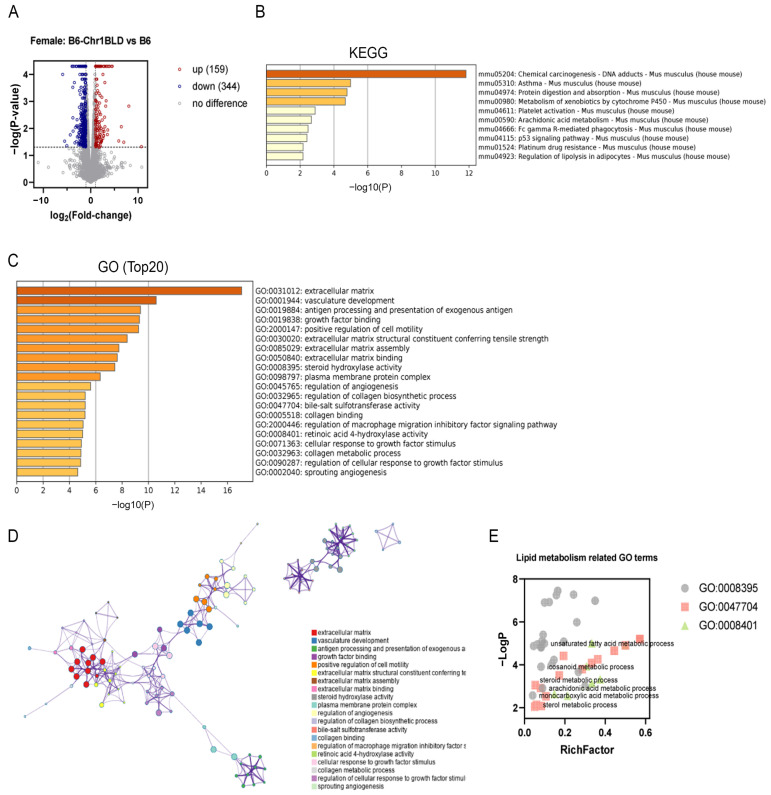
Transcriptome analysis of livers from female B6-Chr1BLD mice. (**A**) Volcano plot of DEGs from female B6-Chr1BLD mice and female B6 mice. (**B**) Enriched KEGG terms. (**C**) Top 20 enriched GO terms. (**D**) Enriched ontology clusters colored by cluster ID. (**E**) Lipid metabolism-related GO terms.

We noticed that the male B6-Chr1BLD line showed pronounced hyperlipidemia phenotypes while females did not, though they had the same genomic variations on Chr 1. We further explored the transcriptomics of female B6-Chr1BLD mice in order to identify the importance of genes between the two genders. Though female B6-Chr1BLD mice showed almost no significant differences in metabolism-related phenotypes compared with female control mice (Figure 4), transcriptomics analysis of female B6-Chr1BLD mice showed a little difference. The volcano plot showed that a total of 503 DEGs were identified, including 159 upregulated genes and 344 down-regulated genes (Figure 7A). KEGG analysis showed that a total of 10 pathways were significantly enriched by DEGs (Figure 7B). Pathways associated with lipid metabolism (arachidonic acid metabolism and regulation of lipolysis in adipocytes) were enriched. GO analysis showed that DEGs were enriched in lipid metabolism-related terms such as unsaturated fatty acid metabolic process, icosanoid metabolic process, arachidonic acid metabolic process, monocarboxylic acid metabolic process and steroid metabolic process (Figure 7C–E). 

**Figure 8:**
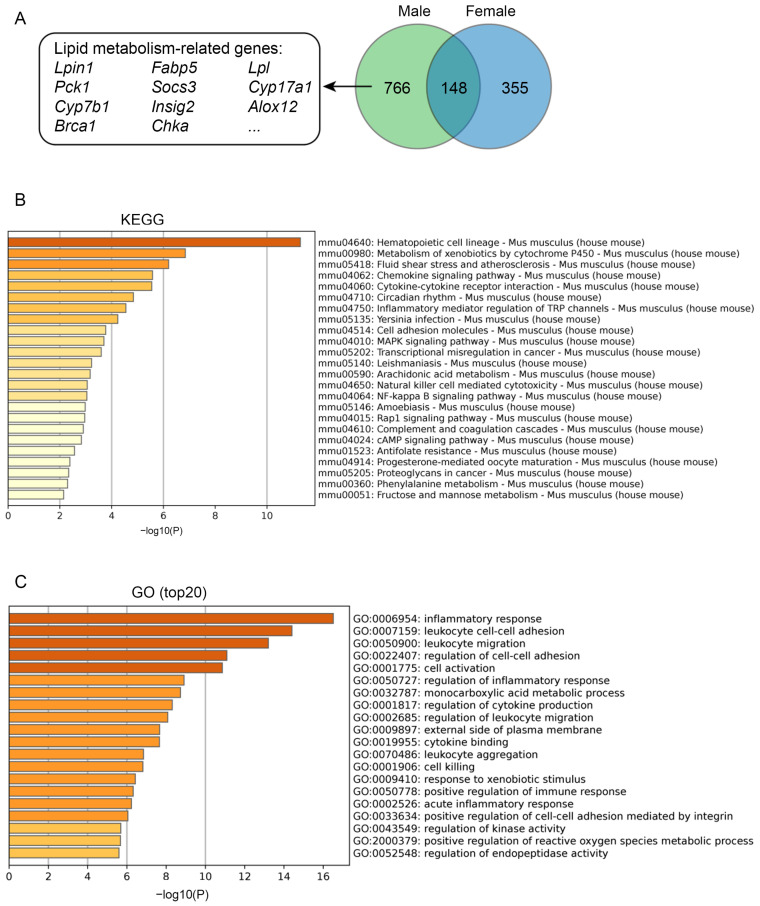
Comparative analysis of the liver transcriptomes of male and female B6-Chr1BLD mice. (**A**) A Venn diagram showing the overlap between DEGs of male and female B6-Chr1BLD mice vs. B6 mice. Partial lipid metabolism-related genes, which are only from male DEGs, are shown. (**B**) Enriched KEGG terms by genes that are only from male DEGs. (**C**) Top 20 enriched GO terms by genes which are only from male DEGs.

We supposed that the differences in metabolic phenotypes between female and male B6-Chr1BLD mice might be mainly due to genes that are differentially expressed only in male mice. A Venn diagram was used to show the differentially expressed gene (Figure 8A). We found 11 lipid metabolism-related genes, including *Lpin1*, *Fabp5*, *Lpl*, *Pck1*, *Socs3*, *Cyp17a1*, *Cyp7b1*, *Insig2*, *Alox12*, *Brca1* and *Chka* [83,84,85,86,87,88,89,90,91,92,93,94]. KEGG analysis showed that a total of 24 pathways were significantly enriched by DEGs (Figure 8B). Pathways associated with lipid metabolism (fluid shear stress and atherosclerosis, and arachidonic acid metabolism) were enriched. GO analysis showed that in the top 20 GO terms, DEGs were enriched in mostly immune-related terms and some lipid metabolism-related terms, such as monocarboxylic acid metabolic process (Figure 8C). Compared with female HFD-fed B6-Chr1BLD mice, the altered expression of lipid metabolism-related genes might cause a more significant hyperlipidemia phenotype in male mice, and the related mechanism needs to be further verified. In conclusion, the phenotypic differences between the two genders in the HFD-fed B6-Chr1BLD line might result from the abnormal typic lipid metabolism pathway.

The phenotypic differences between female and male mice might be sex-dependent, and many studies have reported that estrogens affect lipid metabolism [95,96,97]. Furthermore, many clinical data have also proved that the level of lipid metabolism is different between male and female patients with hyperlipidemia [98,99]. And hyperlipidemia-related drugs have different effects on male and female patients and animal models [100,101]. Based on the clinical differences between male and female hyperlipidemia patients, we need to construct appropriate female or male hyperlipidemia mouse models separately for the study of the disease. Our data revealed that the male B6-Chr1BLD line might serve as a model for male hyperlipidemia patients in the future. Furthermore, in the HFD-fed B6-Chr1SJ line, B6-Chr1LY line and B6-Chr1ZZ2 line, the female mice showed more severe hyperlipidemia phenotypes than male mice. It suggested that these female Chr1 substitution lines might serve as important disease models for female patients. Female patients have a higher incidence rate in older age than men [49,50]. Therefore, we need to identify the hyperlipidemia phenotype in older mice to explore the model potential of these lines for female hyperlipidemia patients. 

Hyperlipidemia is mediated by polygenes. The different hyperlipidemia-related phenotypes between Chr 1 substitution lines and control mice are mainly related to the multigene variants on Chr 1 (Figure 5 and Figure 6). Chr1 substitution lines could be useful for screening variants of hypercholesterolemia. However, there are still several limitations of these Chr 1 substitution lines. Firstly, these Chr 1 substitution lines only contain variants in Chr 1. The variants in other chromosomes also could affect lipid metabolism. Secondly, the source and numbers of the experimental mice were not sufficient. Chromosome 1 of the substitution line was replaced by wild mice from only four different regions of China. Different environments may cause diverse SNP sites and differentially expressed genes on chromosome 1, which might induce different blood lipid-related phenotypes. These lines cannot contain all variants of hypercholesterolemia.

A clear genetic background is important to uncover molecular mechanisms and drug screening. The specific molecular mechanisms underlying the Chr1 substitution lines need to be further investigated, mainly including the transcription levels and protein levels of the genes involved in lipid metabolism, protein interaction and the activation of metabolic and immune pathways. Those data will help to identify the hyperlipidemia-associated variants and clarify the genetic background.

Since the Chr1 of these lines is different from C57BL/6 mice, it will be difficult to mate with gene-engineering mice. After hybridization, homogenous recombination will happen on Chr1. It is a huge work to analyze the recombined Chr1. The better way directly does gene engineering on these Chr1 substitution lines.

Hyperlipidemia can be generally classified as primary and secondary. Primary hyperlipidemia derives from genetic disorders which result from single or multiple gene mutations. Secondary dyslipidemia typically originates from an unhealthy diet with excessive calorie intake and other causes such as hypothyroidism and diabetes [102]. Although LDL- or ApoE-deficient mice are widely used in biomedical research, they do not adequately reflect human disease. These monogenic models result in extreme hyperlipidemia, even neonatal death, and weaken the value of other genes, while common mild-to-moderate hyperlipidemia is typically multigenic [103,104]. Moreover, LDL- or ApoE-deficient mice do not respond well to several clinically used lipid-lowering drugs, which reduces their usability for drug development [105,106]. Our wild mice Chr 1 substitution lines contain multigenic variations in Chr1 from wild mice (Figure 5), which are naturally selected and could better mimic humans. Compared to HFD-induced hyperlipidemia models, our Chr 1 substitution lines showed more significant phenotypes of hyperlipidemia at an earlier stage (Figure 1, Figure 2, Figure 3 and Figure 4), which might help shorten the experimental period. Taken together, Chr 1 substitution lines might be the novel, polygenic interaction-based hyperlipidemia mouse models in the future. This study will provide an animal model basis for new drug research, accurate clinical diagnosis, and precise medicine, which would play an important role in the treatment of hyperlipidemia patients.

## 5. Conclusions

**Figure 9:**
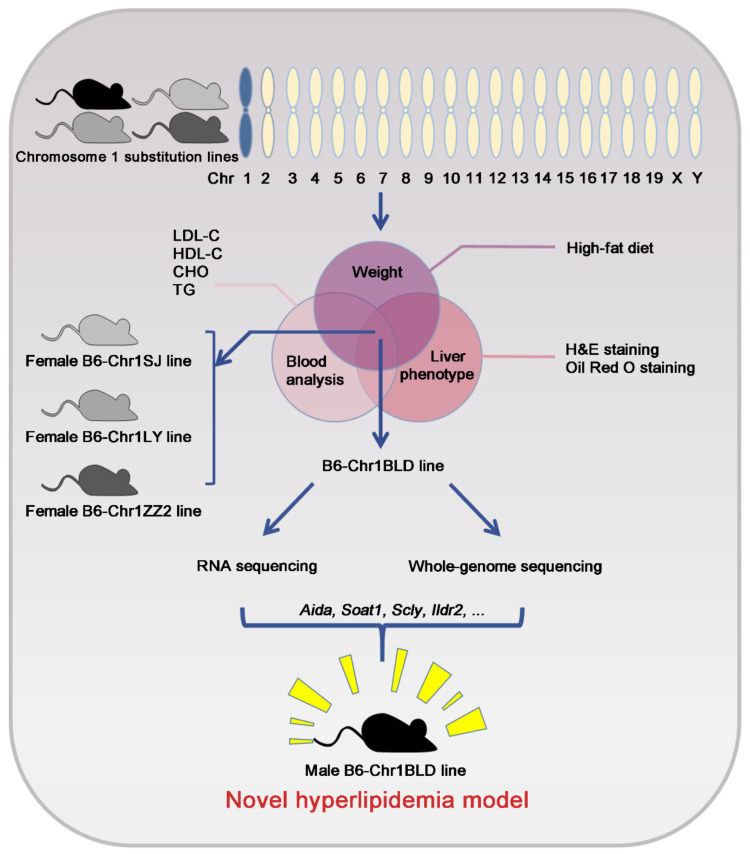
Identification of chromosome 1 substitution lines as novel hyperlipidemia models *via* phenotyping screening.

In conclusion, we screened four Chr 1 substitution lines that contain Chr 1 of wild mice from different regions of China, including B6-Chr1SJ, B6-Chr1LY, B6-Chr1ZZ2 and B6-Chr1BLD. Firstly, we explored the hyperlipidemia-related phenotypes, including body weight, blood analysis and liver pathological phenotypes. The female HFD-fed B6-Chr1SJ line, B6-Chr1LY line, B6-Chr1ZZ2 line and the male HFD-fed B6-Chr1BLD line had a significant hyperlipidemia phenotype. We found the body weight, the levels of inflammatory infiltration and lipid accumulation in liver tissues and the release of lipid metabolism-related factors in the serum of these lines were significantly higher than in control mice. To explore the changes in the signaling pathways, we performed RNA sequencing and whole-genome sequencing of the B6-Chr1BLD line. The results suggested that the male B6-Chr1BLD mice showed differences in DEGs, and SNPs enriched genes in lipid metabolism pathways compared with male C57BL/6J mice, and the main lipid metabolism-related genes, including *Aida*, *Soat1*, *Scly* and *Ildr2* might be involved in the pathologic process of male hyperlipidemic B6-Chr1BLD mice. Taken together, the female B6-Chr1SJ line, B6-Chr1LY line, B6-Chr1ZZ2 line and the male B6-Chr1BLD mice might be the novel, polygenic interaction-based hyperlipidemia mouse models in the future. Our study will provide an animal model basis for new drug research, accurate clinical diagnosis, and precise medicine (Figure 9).

## Data Availability

The data sets used in the current study are available from the corresponding author upon reasonable request.
